# A High-Content, Multiplexed Screen in Human Breast Cancer Cells Identifies Profilin-1 Inducers with Anti-Migratory Activities

**DOI:** 10.1371/journal.pone.0088350

**Published:** 2014-02-10

**Authors:** Marion E. Joy, Laura L. Vollmer, Keren Hulkower, Andrew M. Stern, Cameron K. Peterson, R. C. “Dutch” Boltz, Partha Roy, Andreas Vogt

**Affiliations:** 1 Department of Bioengineering, University of Pittsburgh, Pittsburgh, Pennsylvania, United States of America; 2 Department of Computational and Systems Biology, University of Pittsburgh, Pittsburgh, Pennsylvania, United States of America; 3 Department of Pathology, University of Pittsburgh, Pittsburgh, Pennsylvania, United States of America; 4 University of Pittsburgh Drug Discovery Institute, Pittsburgh, Pennsylvania, United States of America; 5 Magee Women's Research Institute, Pittsburgh, Pennsylvania, United States of America; 6 Platypus Technologies, LLC, Madison, Wisconsin, United States of America; Vanderbilt University Medical Center, United States of America

## Abstract

Profilin-1 (Pfn-1) is a ubiquitously expressed actin-binding protein that is essential for normal cell proliferation and migration. In breast cancer and several other adenocarcinomas, Pfn-1 expression is downregulated when compared to normal tissues. Previous studies from our laboratory have shown that genetically modulating Pfn-1 expression significantly impacts proliferation, migration, and invasion of breast cancer cells *in vitro*, and mammary tumor growth, dissemination, and metastatic colonization *in vivo*. Therefore, small molecules that can modulate Pfn-1 expression could have therapeutic potential in the treatment of metastatic breast cancer. The overall goal of this study was to perform a multiplexed phenotypic screen to identify compounds that inhibit cell motility through upregulation of Pfn-1. Screening of a test cassette of 1280 compounds with known biological activities on an Oris™ Pro 384 cell migration platform identified several agents that increased Pfn-1 expression greater than two-fold over vehicle controls and exerted anti-migratory effects in the absence of overt cytotoxicity in MDA-MB-231 human breast cancer cells. Concentration-response confirmation and orthogonal follow-up assays identified two bona fide inducers of Pfn-1, purvalanol and tyrphostin A9, that confirmed in single-cell motility assays and Western blot analyses. SiRNA-mediated knockdown of Pfn-1 abrogated the inhibitory effect of tyrphostin A9 on cell migration, suggesting Pfn-1 is mechanistically linked to tyrphostin A9′s anti-migratory activity. The data illustrate the utility of the high-content cell motility assay to discover novel targeted anti-migratory agents by integrating functional phenotypic analyses with target-specific readouts in a single assay platform.

## Introduction

Tumor metastasis is a complex series of events, during which cells disseminate from the primary tumor, enter the circulation, extravasate, and colonize target tissues [1]. Cell motility plays a vital role in at least some of these events. Consequently, agents that inhibit cell motility could be beneficial in the treatment of metastatic cancers, and anti-migratory or anti-invasive activity is usually viewed as a desired attribute for novel anticancer drugs [2].

**Figure 1:**
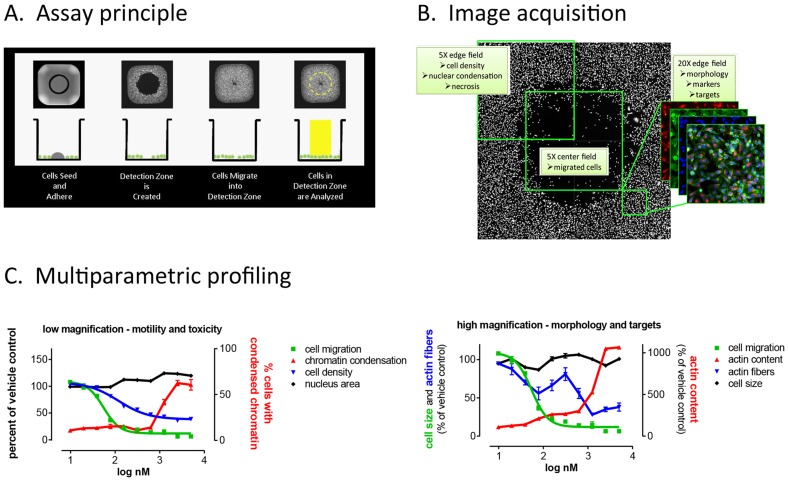
A high-throughput cell motility assay that enables multiplexed image-based analysis of cell migration and associated pharmacodynamic markers. A. Schematic of Oris™ Pro 384 Cell Migration Assay. Cells are seeded and allowed to adhere in an annular monolayer surrounding a Biocompatible Gel (BCG). The BCG dissolves to reveal a cell-free Detection Zone into which cells migrate. B. Wells are imaged via microscopy or High Content Imagers, and images analyzed for cell migration as well as phenotypic changes. C. Quantitation of readouts enables assembly of compound activity profiles including multiparameter toxicity (*left panel*), cell morphology, and pharmacodynamic markers of compound activity (*right panel*), exemplified by the actin-depolymerizing agent, cytochalasin D. All readouts are correlated with the primary functional phenotypic readout (*cell migration*).

The majority of contemporary drug discovery efforts are based on high-throughput screening (HTS). The principal barrier to performing HTS to discover anti-migratory agents is the lack of assays that are robust, reproducible, and compatible with the demands of HTS. Cell-based assays in 384-well plates are commonly performed but none are formatted for cell migration studies. Likewise, cell migration assays exist in many formats (for a recent review see [3]) but they are either not suited for automation (Boyden chambers), require manual processing steps [4]–[6], or do not allow free and open access to wells for imaging (Roche xCELLigence system), thereby eliminating the possibility to perform simultaneous measurements of cell motility and associated target- or pathway-specific biomarkers. Here we describe the HTS implementation and validation of a novel, multiparametric cell migration assay that does not require mechanical processing and that is fully compatible with automated microscopy and high-content screening (Oris™ Pro, Platypus Technologies) (**Figure 1**). Using this platform, we screened a test cassette of small molecules with known biological activities (Library of Pharmacologically Active Compounds, LOPAC) and identified six compounds that selectively inhibited breast cancer cell migration, all of which had targets associated with cell motility. The assay had a 83% confirmation rate in concentration-response; two agents further confirmed in single-cell motility studies.

We then exploited the assay's imaging compatibility to identify compounds that perturb expression of Pfn-1 in breast cancer cells. Pfn-1 is a ubiquitously expressed actin-monomer binding protein and an essential regulator of actin polymerization in cells, which has been shown to be an obligatory molecular player for actin-based cell motility in almost all physiological contexts [7]–[13]. Seemingly counter-intuitive to the essential role of Pfn-1 in cell migration in physiological contexts, invasive and metastatic breast cancer cells present with downregulation of Pfn-1 expression and in fact, Pfn-1 depletion can promote migration and invasion of metastatic human breast cancer cell lines *in vitro* and escape from the primary tumor *in vivo* [14]. Conversely, overexpression of Pfn-1 inhibits proliferation, migration, and invasion of breast cancer cells *in vitro* and suppresses tumor growth *in vivo* [15]–[18]. Agents that induce Pfn-1 would thus be expected to exert an anti-migratory phenotype. The multiplexed motility assay identified two compounds that induced Pfn-1 greater than two-fold over vehicle-treated controls and elicited anti-migratory activity in human breast cancer cells, and one of these agents further showed functional involvement of Pfn-1 in its anti-migratory action, providing biological validation of the analytical approach. The data illustrate the utility and flexibility of the Oris™ Pro cell migration assay as a unique and versatile tool to discover anti-migratory agents with defined cellular target activities.

## Results

### HTS assay development and implementation

**Figure 2:**
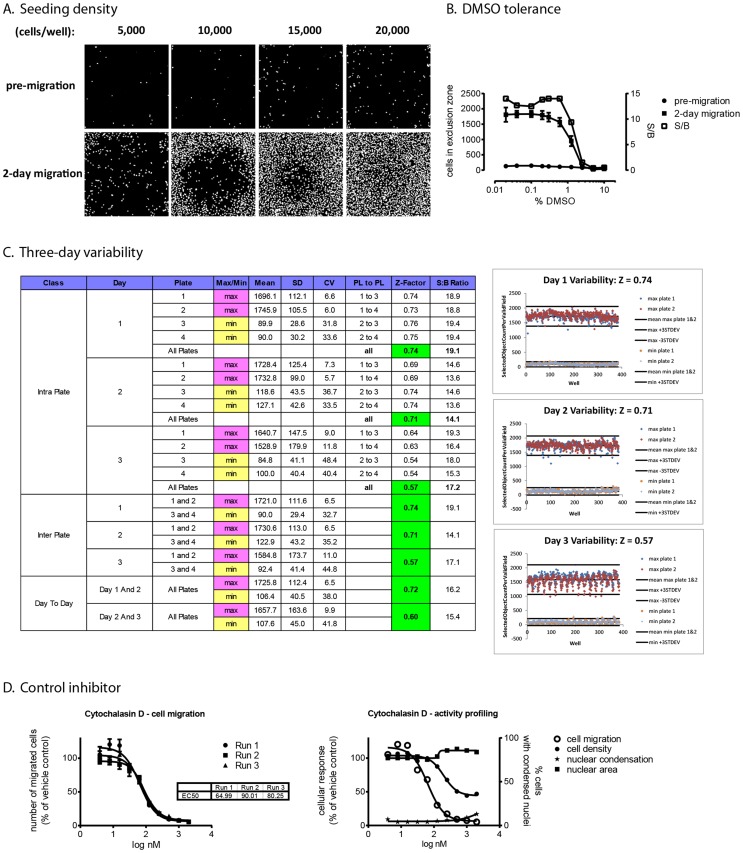
HTS assay development. MDA-MB-231 cells were plated in Oris™ Pro 384 plates and allowed to attach for 2 h. Plates were stained with Hoechst 33342 immediately thereafter (*pre-migration*) or after 2 days in culture (*2-day migration*), and imaged on the ArrayScan II. **A. Seeding density.** Optimal gap closure with minimal background was obtained at 15,000 cells/well. **B. DMSO tolerance.** 16 wells each of minimum (pre-migration) and maximum (two-day migration) controls were treated with a ten-point, two-fold gradient of vehicle (DMSO) and numbers of cells that had migrated into the exclusion zone were enumerated. Assay performance decreased at concentrations above 0.6% DMSO due to toxicity. **C. Three-day variability.** Two full microplates of minimum and maximum controls were treated with vehicle (0.1% DMSO) on three consecutive days using equipment to be used in HTS. Intra-plate and inter-plate variability parameters were calculated (*Table*). SD, standard deviation; CV, coefficient of variance; PL to PL, plate to plate comparison; S:B ratio, signal-to-background ratio. Scatter plots illustrate day to day performance; the lower Z-factor on day 3 was a result of a partially obstructed dispense manifold. **D. Control inhibitor studies.** Using optimized assay conditions, identical IC_50_ curves for cytochalasin D were obtained in three independent runs (*left panel*). Multiparametric profiling of cell migration, toxicity (cell density), and nuclear morphology (brightness and area) document selective inhibition of cell migration in the absence of overt cytotoxicity (*right panel*).

The Oris™ Pro 384 cell migration assay was optimized and validated according to universally accepted performance and reproducibility criteria [19]. We chose MDA-MB-231 as a model for human breast cancer cells because this metastatic cell line exhibits highly motile characteristics in culture. Preliminary experiments documented a plating volume of 15 µl and a migration time of 48 h to be optimal for this cell line (data not shown). **Figure 2A** shows that under these conditions, gap closure was most robust at 15,000 cells/well without signs of overcrowding or “break-through” of cells into the exclusion zone during cell attachment. Using the number of cells in the exclusion zone at the beginning (“pre-migration”) and the end of the study (“2-day migration”), preliminary assay performance measurements indicated signal-to-background ratios of >10 and Z-factors above 0.6 (data not shown). Based on these results, a seeding density of 15,000 cells was chosen for all further development studies. The assay tolerated up to 0.6% DMSO, after which assay performance decreased due to cellular toxicity (**Figure 2B**). We then implemented the assay on HTS equipment for multi-day variability studies. Two full 384-well microplates of minimum (pre-migration) and maximum (2-day migration) controls were prepared on three consecutive days, and intra-plate and inter-plate variability assessed as previously described [20]. **Figure 2C** documents HTS performance on all three days. There were no process errors on day 1 and 2; Z-factors were 0.74 and 0.71, respectively. The repeating pattern of lower cell numbers in some wells on day 3 was a result of a partially obstructed dispense manifold on the automated liquid handler that did not affect assay performance (Z-factor 0.57). Inter-plate and intra-plate variability was <10% for the maximum controls. The higher CVs seen with the pre-migration controls are an artifact that is common with very low values [19]; this did not affect HTS performance. IC_50_s of the control inhibitor, cytochalasin D, were identical on three consecutive days; multiparametric measurements of cell migration and cytotoxicity confirmed selective inhibition of cell motility in the absence of overt toxicity (**Figure 2D**). Thus, the assay met universally accepted HTS criteria and was moved into HTS validation studies.

### Assay validation

**Figure 3:**
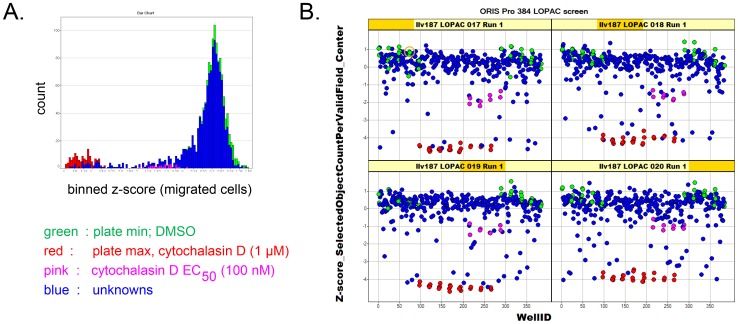
LOPAC library screening for inhibitors of cell migration. Cells were treated in duplicate Oris™ Pro 384 plates with vehicle (*green*), 1 µM cytochalasin D (*red*), 0.1 µM cytochalasin D (*pink*), *or* 10 µM of compounds (*blue*) for 2 days. Cells that migrated into the exclusion zone were enumerated by high-content analysis on the ArrayScan II. **A. Histograms** show that positive and negative controls were well separated and data largely followed a normal distribution. **B. Trellis plots.** Z-scores were calculated for each data point based on plate average and plotted against well number. Data are from one replicate run; the y-axis shows z-scores of migrated cells.

We screened the 1280 member Library of Pharmacologically Active Compounds (LOPAC) for inhibition of cell migration under optimized assay conditions, using image acquisition and analysis on the ArrayScan II as described earlier [21] and detailed in Materials and Methods. The library screen consisted of four plates run in duplicate; the primary hit selection parameter was the number of cells that migrated into the exclusion zone. **Figure 3A** shows that negative (DMSO, green) and positive (1 µM cytochalasin D, red) intra-plate controls were well separated; the average Z-factor for all eight plates was 0.72. Data followed a normal distribution. As expected, the screen identified a number of compounds that reduced cell migration (**Figure 3B**). A hit criterion was set as z-score (migrated cells)<−3, which selected a total of 47 compounds (a 3.7% hit rate) (**Table S1**).

### Hit confirmation

The main confounding factor in bulk cell migration assays is cellular toxicity. Therefore, we imaged cells in an area of the well not affected by cell migration (see image acquisition scheme in **Figure 1**), which permitted measurements of cell loss and changes in nuclear morphology during the primary screen. The initial set of 47 agents that inhibited cell migration with z-scores<−3 was enriched for known cytotoxic agents and nuisance compounds (**Table S1, marked in yellow**). While it is likely that some of those agents also possess anti-migratory properties, their overt toxicity obscured motility measurements, and we therefore focused on a subset of compounds with low to moderate toxicity (≤40% cell loss compared to vehicle control). This primary hit selection paradigm identified nine compounds that selectively inhibited cell migration. Two compounds (indatraline and bromoacetyl alprenolol menthane) did not repeat with the same level of statistical significance in both runs. Six compounds were commercially available and were repurchased for concentration-response confirmation. All had cellular targets associated with cell migration (**Table 1**).

**Table 1:** Prioritized cell migration inhibitors from the LOPAC screen.

Compound name	Targets^a^	References linking targets to cell migration
GW5074	c-Raf1	[29]
Tyrphostin AG 879	TrkA, Her2	[30]
7-Cyclopentyl-5-(4-phenoxy)phenyl-7H-pyrrolo[2,3-d]pyrimidin-4-ylamine	src-family kinases	[31]
GR 127935 hydrochloride	5-HT1B/1D receptors	[32], [33]
3′,4′-Dichlorobenzamil	Na+/Ca2+ exchanger	[34]
Dihydroouabain	Na+/K+ ATPase	[35]

a according to SIGMA-Aldrich LOPAC description.

### Concentration-response confirmation

**Figure 4:**
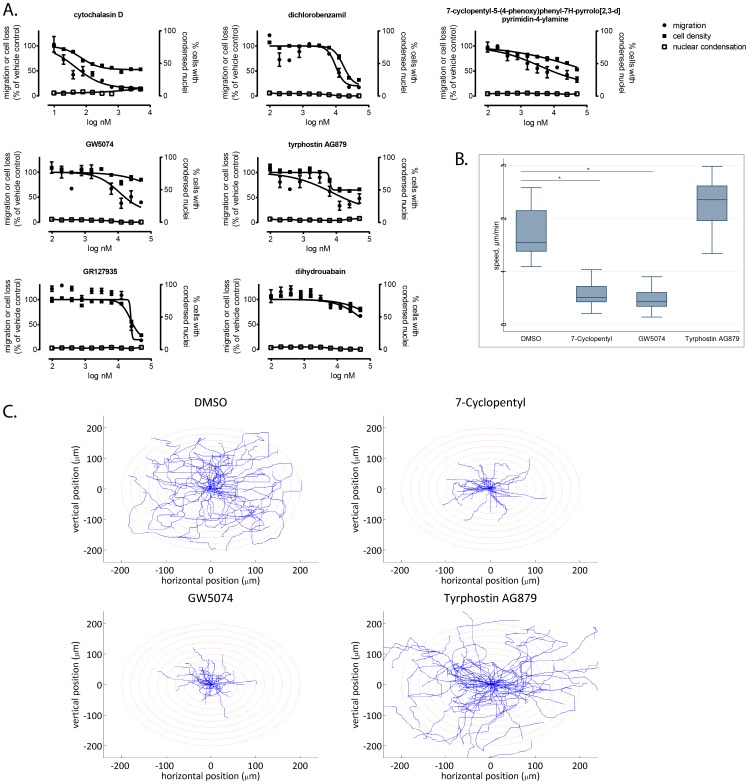
Confirmation of positives from the high-content cell migration screen. A. Multiparameter concentration-response confirmation in the Oris™ Pro assay. MDA-MB-231 cells were treated for 48 h with ten point, two-fold concentration gradients of test agents. Cells were stained with Hoechst 33342 and analyzed for cell migration, cell density, and condensed nuclei as described in Materials and Methods. Data are the averages ± SE from quadruplicate determinations and are from a single experiment that has been repeated twice. B. and C. Single-cell motility assay. Agents that showed selective, concentration-dependent inhibition of cell migration in the primary assay format were tested in a single-cell motility assay. Two compounds significantly reduced cell migration velocity compared with vehicle control. Box, 25th and 75th percentiles, whiskers, 10th and 90^th^ percentiles; line, median. Data are the combined values from two independent experiments, each comprising 28 individual cells. Rose plots in C. illustrate motility patterns of individual cells. Each line represents the trajectory of a single cell over a period of 48 h.

The six repurchased agents were tested in ten-point, two-fold concentration-response studies for inhibition of cell migration and cytotoxicity using the primary assay. Three (GW5074, tyrphostin AG879, and 7-cyclopentyl-5-(4-phenoxy)phenyl-7H-pyrrolo[2,3-d]pyrimidin-4-ylamine)) confirmed in concentration-response and showed selective inhibition of cell migration (**Figure 4A**). Two (GR127935 and dichlorobenzamil) showed concentration-dependent inhibition of cell migration but also nonselective toxicity. Only one agent (dihydrouabain) did not confirm. None of the agents tested had elevated numbers of cells with condensed nuclei. Single-cell motility analysis confirmed the anti-migratory activities of two compounds, GW5074 and 7-cyclopentyl-5-(4-phenoxy)phenyl-7H-pyrrolo[2,3-d]pyrimidin-4-ylamine) (**Figure 4B and 4C**). Thus, the concentration-response studies validated the ability of the Oris™ Pro assay to discover agents with bona fide anti-migratory activity and to distinguish anti-migratory from antiproliferative or apoptotic activities.

### HCS extension of cell migration screen

We then took advantage of the assay's compatibility with automated imaging and high-content analysis and interrogated the LOPAC library for compounds that increase Pfn-1 expression. The identical plates from the primary migration screen were immunostained with an anti-Pfn-1 antibody, and average Pfn-1 staining intensity per cell was quantified and correlated to anti-migratory activity. Because the Pfn-1 expression data set was not normally distributed and lacked a validated small molecule positive control (**Figure S1**), we chose a controls-based hit selection criterion. We identified 32 agents (2.5%) that elevated Pfn-1 more than 2-fold over vehicle controls. As was the case for the cell migration screen, this set was enriched for known cytotoxic substances, including many clinically used antineoplastic agents, which were eliminated based on cell density measurements as for the cell migration screen. Of the 12 remaining compounds, four (purvalanol, tyrphostin A9, 5-azacytidine, and indirubin-3-oxime) were selected for concentration-response confirmation because they repeated in both runs and appeared to have elevated levels of Pfn-1 based on visual inspection of archived scan images (**Figure S1**). All four had anti-migratory activities in the primary cell migration screen and targets associated with cell motility (**Table S1**).

### Hit confirmation

**Figure 5:**
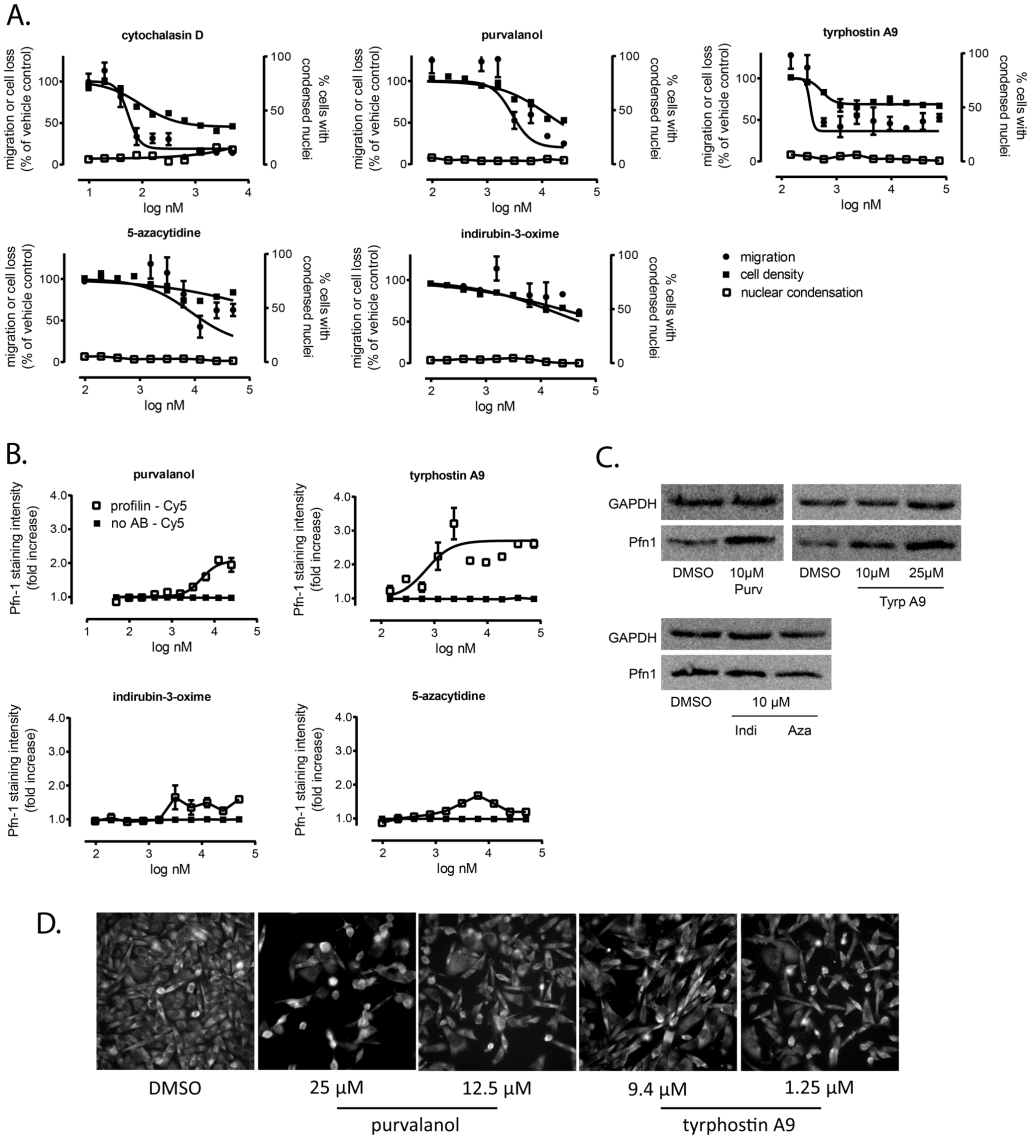
Identification of agents that increase Pfn-1 expression. Compounds that emerged as positives from the Pfn-1 expression counterscreen were treated with ten-point, two-fold concentration gradients of test agents and analyzed for cell migration, cytotoxicity, and Pfn-1 induction. **A. Multiparametric analysis** of antimigratory activity and toxicity. **B. Pfn-1 expression.** Concentration-response plates from **A.** were immunostained with a Pfn-1/Cy5 secondary antibody pair (open squares) and analyzed for Pfn-1 levels by high-content analysis. Staining specificity was determined in the absence of primary antibody (closed squares). Data in **A**. and **B.** are the averages ± SE from quadruplicate determinations and are from a single experiment that has been repeated twice. **C. Western blot** analysis confirming elevated Pfn-1 expression by purvalanol and tyrphostin A9. **D. Representative fluorescence micrographs** of the two confirmed positives from the Pfn-1 induction screen in an area of the well adjacent to the exclusion zone.

All four positives from the Pfn-1 screen were tested in ten-point, two-fold concentration-response assays using a multiparametric assay design encompassing cell migration, toxicity, nuclear morphology, and Pfn-1 expression. All primary positives except indirubin-3-oxime inhibited cell migration in the absence of overt cytotoxicity (**Figure 5A**).

Whereas all four positives from the Pfn-1 induction screen appeared to have visibly increased Pfn-1 expression (**Figure S1**), only tyrphostin A9 and purvalanol showed robust, concentration-dependent increases in Pfn-1 expression when analyzed by high-content analysis (**Figure 5B**). These results were confirmed by Western blot analysis where tyrphostin A9 and purvalanol increased Pfn-1, whereas 5-azacytidine and indirubin-3-oxime did not (**Figure 5C**). Selected fluorescence micrographs of purvalanol or tyrphostin A9 treated cells at intermediate (1.25 µM for tyrphostin A9 and 12.5 µM for purvalanol) and maximal antimigratory concentrations (9.4 µM for tyrphostin A9 and 25 µM for purvalanol) confirmed a mild but discernible increase in Pfn-1 immunoreactivity (**Figure 5D**). Cytochalasin D also showed concentration-response by immunofluorescence, but did not confirm by Western blot (data not shown). This suggests that the assay is sensitive to morphological artifacts, necessitating the need for non-image-based confirmatory assays. Because the assay scores cell migration based on number of cells, which could be confounded by cell cycle arrest, we performed both cell cycle analysis and single-cell motility assays to validate the compounds' anti-migratory activities. For cell cycle analysis, DNA content was measured in 4,000 individual cells imaged on the Arrayscan II. The only agent that appreciably changed cell cycle distribution was purvalanol, which increased the number of cells in G2/M (**Figure S2**), consistent with its ability to inhibit CDK activity. In contrast, tyrphostin A9 and cytochalasin D did not appreciably alter cell cycle distribution compared with vehicle control.

**Figure 6:**
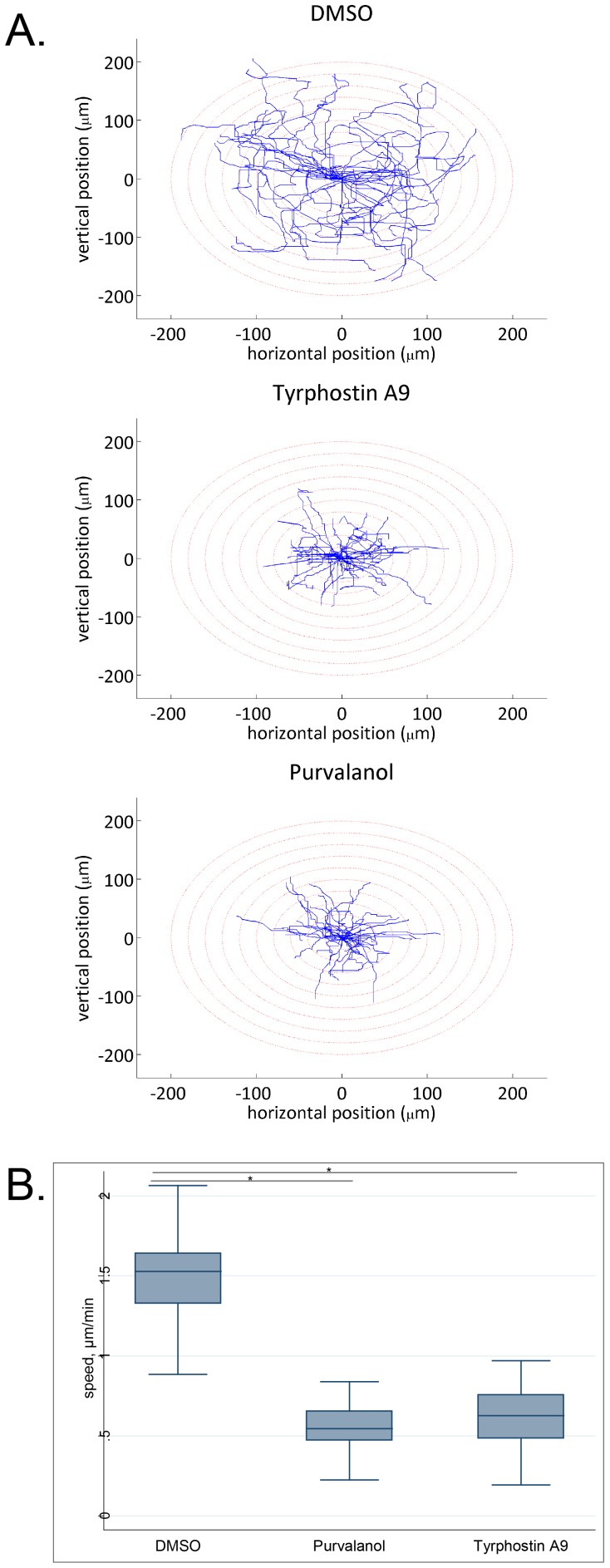
Confirmation of anti-migratory activity by Pfn-1 inducing agents. Cells were treated with compounds for 48-1 coated cell culture dishes, replated and imaged by time-lapse videomicroscopy. **A. Trajectories of individual MDA-MB-231 cells** of different experimental groups in time-lapse motility assay. Data are from a single experiment that has been repeated once with identical results. **B. Box and whisker plots** representing the average speed of migration of MDA-MB-231 cells treated with DMSO (control) vs. 10 µM of either purvalanol or tyrphostin A9. Box, 25th and 75th percentiles; whiskers, 10th and 90th percentiles; line, median. Data are pooled cell data from two independent experiments (n = 20 cells per group; *p<0.001).

**Figure 7:**
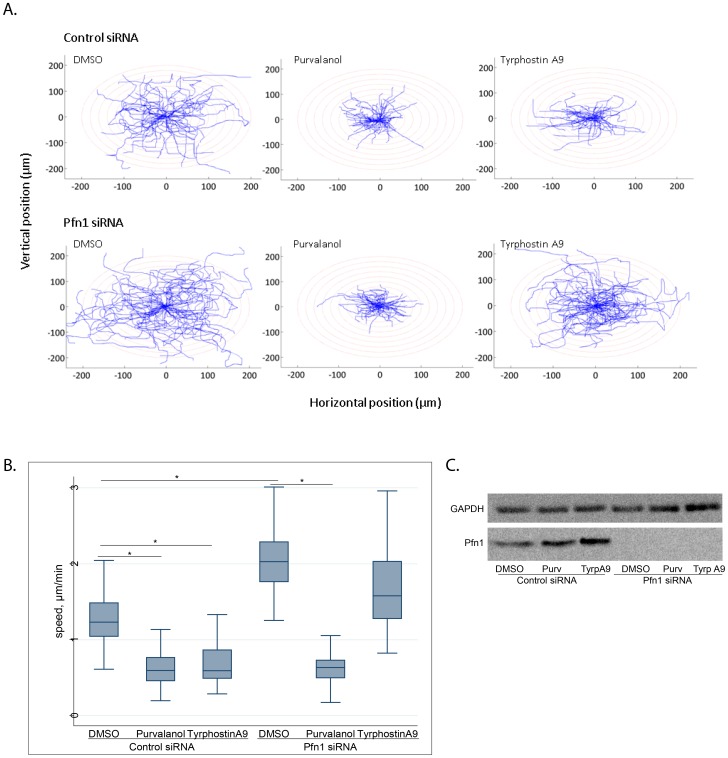
Pfn-1 knockdown abolishes anti-migratory activity of tyrphostin A9 but not purvalanol. A. Trajectories of individual cells treated with vehicle (DMSO), 10 µM purvalanol, or 10 µM tyrphostin A9. **B. Box and whiskers plot** documenting significant reduction in cell motility by both agents and reversal of anti-migratory activity by Pfn-1 siRNA of tyrphostin A9 but not purvalanol. Data are pooled from two independent experiments comprised of 28 cells each. *, p<0.0001. **C. Western blot** analysis of Pfn-1 expression in the presence or absence of Pfn-1 siRNA. Data are from a single experiment that has been repeated twice with identical results.

We next confirmed the anti-migratory phenotypes of purvalanol and tyrphostin A9 in a single-cell motility assay and found that at the concentration used in the primary screen, both agents significantly reduced cell motility compared with vehicle control (**Figure 6**). Based on our previous findings of Pfn-1′s inhibitory effect on collagen invasiveness of MDA-MB-231 cells [14], [15], we also confirmed that both purvalanol and tyrphostin A9 significantly reduced collagen invasiveness of MDA-MB-231 cells (**Figure S3**). Finally, we asked whether Pfn-1 was functionally involved in the anti-migratory activities of purvalanol and tyrphostin A9. If their anti-migratory effects involved the action of Pfn-1, one would expect that their activity should be abrogated or at least substantially diminished upon Pfn-1 depletion. We therefore performed single-cell motility assays to compare the effects of tyrphostin A9 and purvalanol on cell motility in Pfn-1-proficient vs. -depleted conditions. **Figure 7** shows that in the presence of control siRNA, both purvalanol and tyrphostin A9 significantly reduced cell motility, as expected. When Pfn-1 was knocked down, DMSO-treated cells migrated faster, consistent with our previously published data [22], [23]. Importantly, knockdown of Pfn-1 abolished the anti-migratory activity of tyrphostin A9 but not of purvalanol (**Figure 7**). Western blots confirmed elevated levels of Pfn-1 after compound treatments with control siRNA but not Pfn-1 siRNA (**Figure 7C**). Taken together, the results suggest Pfn-1 is mechanistically linked to cell migration inhibition by tyrphostin A9, providing biological validation to the analytical approach.

## Discussion

Cancer metastasis represents a dire unmet medical need. Ninety percent of cancer-related deaths occur by metastasis, but effective therapies are lacking. Cell motility plays a critical role in the metastatic process; therefore agents that inhibit cell motility could find application as novel antimetastatic agents. The discovery of such agents is critically dependent on cellular assays that are high-throughput and recapitulate at least some aspects of the metastatic cascade. In this report, we have developed and HTS implemented an innovative cell-based migration assay that is compatible with automated microscopy, thereby enabling the correlation of a biological endpoint with cellular toxicity and specific target activities.

The assay, which does not require mechanical processing steps, was implemented on laboratory automation equipment and satisfied universally accepted HTS performance criteria. Multiparametric screening of a library of compounds with known biological activities revealed many agents that inhibited cell migration. All primary hits had targets associated with cell motility, and some of the positives (i.e., purvalanol and indirubin-3-oxime) had been found in a prior cell invasion screen [24]. We decided to prioritize and pursue compounds that selectively inhibited cell migration in the absence of overt toxicity. While this strategy was chosen to document the assay's ability to distinguish between the two biological activities, in a discovery screen this could result in a large number of false negatives, as many of the cytotoxic agents could be selective inhibitors of cell migration at lower concentrations. This could be overcome by rescreening the library at a lower concentration. Alternatively, the hit criterion could be altered to include cytotoxics into the primary hit identification scheme and to determine their selectivity in concentration-dependence, follow-up assays.

Whereas the assay had a good confirmation rate in concentration-response, some compounds did not repeat in orthogonal assays, such as Western blots and single-cell motility assays. This is likely a result of the imaging assay being sensitive to morphological changes, and underscores the need for non-fluorescence-based counter-assays as critical components of a secondary screening paradigm.

A subset of the anti-migratory hits induced the expression of the small actin–binding protein, Pfn-1. Pfn-1 modulates breast cancer aggressiveness, and genetic overexpression of Pfn-1 reduces tumor formation *in vivo*, and reduces cell migration and invasion in breast cancer cells [14]. Two compounds, purvalanol and tyrphostin A9 (also known as tyrphostin RG 50872 [25]), were confirmed by Western blot and in single-cell motility assays. Both compounds had previously been found to inhibit cell motility [24], [26]. siRNA knockdown experiments revealed that one of them (tyrphostin A9) lost anti-migratory activity in Pfn-1 depleted cells, indicating a functional involvement of Pfn-1 in its anti-migratory activity.

In summary, the multiparametric high-content screen identified two bona fide inducers of Pfn-1 with anti-migratory activities, and for at least one of the agents, Pfn-1 appears to be mechanistically linked to anti-migratory activity. The data highlight the utility of the Oris™ Pro high-content cell motility assay to integrate functional phenotypic analyses with target-specific readouts in a single assay, adding a novel, validated tool to our armamentarium to discover potential antimetastatic agents. A three-dimensional version of the Oris™ Pro assay is currently being validated.

## Materials and Methods

### Cell culture

The MDA-MB-231 breast cancer cell line was from the American Type Culture Collection (ATCC, Manassas, VA) and maintained as described [27]. The identity of the line was confirmed by The Research Animal Diagnostic Laboratory (RADIL) at the University of Missouri, Columbia, MO (http://www.radil.missouri.edu), using a PCR based method that detects 9 short tandem repeat (STR) loci, followed by comparison of results to the ATCC STR database. A cell bank of defined passage was established and cells were propagated for no more than ten passages in culture.

### Compound treatment and sample processing

The Library of Pharmacologically Active Compounds (LOPAC, Sigma-Aldrich) was maintained in assay ready format in a Matrical Ministore under temperature and humidity-controlled conditions. Assays were conducted in collagen-coated Oris™ Pro 384-well microplates (Platypus Technologies, Madison, WI, cat # PRO384CMACC5). The assay uses an innovative design where a biocompatible gel (BCG) is precisely positioned in the center of the wells of a 384-well microplate (**Figure 1A**). Upon cell seeding, the BCG dissolves and reveals an exclusion zone into which cells can migrate. Cell migration can be followed visually or by time lapse microscopy, enabling real-time kinetic measurements. At the end of the migration period, cells can be stained with fluorescent probes or antibody conjugates, and analyzed on high content readers (**Figure 1B**). The multiparametric nature of the assay format permits simultaneous, quantitative measurements of targets and pathways that are correlated to a functional phenotypic readout (**Figure 1C**). Cells were seeded at a density of 1.5×10^4^ in 15 µl complete growth medium and plates centrifuged for 1 min at 50× g. After a 2 h incubation period, medium was removed and cells were washed once with PBS. For compound treatments, plates containing aliquots of compound stock solutions in 100% DMSO were reconstituted to 30 µM in growth medium on the day of experiment. Fifteen µl of 30 µM compound solutions were transferred to assay plates using a Janus MDT automated workstation (PerkinElmer). Each plate contained 32 wells of negative controls (0.1% DMSO), 24 wells of positive controls (1 µM cytochalasin D), and 8 wells of an intermediate concentration of positive control (100 nM cytochalasin D). Forty-eight hours after treatment, cells were fixed with formaldehyde (4%) and stained with Hoechst 33342 (Invitrogen, H1399) at 10 µg/ml in PBS. After a 30 min incubation at room temperature, plates were washed three times with PBS, sealed, and stored at 4°C until imaging. For experiments involving pre-migration controls, plates were fixed and stained immediately after compound treatment. All processing steps except compound treatment were done with a Titertek MAP-C2. For confirmatory studies, compounds were repurchased from Sigma-Aldrich and dissolved in DMSO.

### Imaging and analysis

For the primary cell migration assay, two separate scans were performed on the ArrayScan II (Thermo Fisher Cellomics) at a single wavelength of 350/461 nm (DAPI, Hoechst) using a 5× objective. For cell migration measurements, a single image positioned in the center of the well was acquired; for cell density measurements, a second field was acquired at the edge of the well. Nuclei were detected and quantified by the Target Activation Bioapplication (Thermo Fisher Cellomics), as described [28]. To select hits, data from both runs were averaged and z-scores calculated for each data point. Compounds with z-scores<-3 in the migration screen were selected as primary positives. Percent cell loss was calculated as % toxicity = 1-((cell density_compound_/cell density_DMSO controls_)*100). Positives were assayed for concentration-response in three independent experiments, each using ten point, two-fold concentration gradients of test agents in quadruplicate.

Cell cycle analysis was performed on archived images of Hoechst 33342-stained nuclei. Two to three images, positioned away from the exclusion zone but not touching the well edge were acquired with a 10×0.5 NA objective on the ArrayScan II (see **Figure S2** for an illustration). Total nuclear staining intensity in an area defined by the nuclear mask, enlarged by 2 pixels, was measured in a minimum of 1,000 individual cells per well. ArrayScan tabular data from four pooled wells were converted to flow cytometry format using Text2FCS software (Joseph Trotter, Scripps Research Institute, La Jolla, CA). Cell cycle distributions were estimated by the Dean-Jett-Fox modeling method using the FlowJo software package (Tree Star, Inc., Ashley, OR).

For the Pfn-1 expression screen, microplates from the primary screen were permeabilized for 5 min with 0.2% Triton X-100, blocked with 1% BSA in PBS for 30 min., and incubated with a Pfn-1 antibody (Novus, NBP1-9584) at a 1∶800 dilution. Pfn-1 immunoreactivity was visualized by a Cy3-conjugated secondary antibody (Jackson Immunoresearch, 1∶400 dilution). Three image fields, positioned away from the exclusion zone but not touching the well edge, were acquired on an ArrayScan VTi using a 20× objective and a Cy3 compatible filter set (XF93, Omega Optical) at excitation/emission wavelengths of 350/461 nm (Hoechst) and 556/573 nm (Cy3). A nuclear mask was generated based on Hoechst signal. Pfn-1 expression was quantified by the Compartmental Analysis Bioapplication in an area defined by the nuclear mask, enlarged by 3 pixels to capture cytoplasmic and nuclear Pfn-1 expression. Each data point was normalized to Pfn-1 staining of vehicle controls on a plate-by-plate basis. Agents that increased Pfn-1 greater than two-fold over controls were selected as positives, and assayed for concentration-response in three independent experiments, each using ten point, two-fold concentration gradients of test agents in quadruplicate.

### Time-lapse cell motility assay

MDA-MB-231 cells were treated with each agent at 10 µM working concentration. Twenty-four h following initial treatment, cells were re-plated in collagen-coated 48-well plates for single-cell motility assays, and were again treated with 10 µM compound. The next day, cells were imaged for 3 h at 60 s time intervals between successive image frames. During imaging, the culture plate was placed in an incubation chamber (LiveCell™ System, Pathology Devices Inc.) to maintain appropriate environmental conditions (37°C/pH 7.4). Cell trajectory was generated by frame-by-frame analysis of the centroid positions (x, y) of cell nuclei (assumed to be the representations of cell bodies). 20-40 individual cells were scored in each experiment. All images were acquired and quantified using Metamorph and NIH ImageJ software, respectively.

### Statistics

Statistical analyses were performed using ANOVA, followed by Tukey-Kramer post-hoc test for multiple comparisons. P-values less than 0.001 were indicated as significant. Box and whisker plots were used to represent experimental data (box: 25th and 75th percentile; whisker: 10th and 90th percentile; line: median). All statistical tests were performed with Stata/SE software (StataCorp). 2011. Stata Statistical Software: Release 12. College Station, TX: StataCorp LP.).

### siRNA transfection

For single-cell motility assays and Western blots, positive hits were repurchased from Sigma-Aldrich and diluted in DMSO at 10 mM stock concentrations. Cells were transfected with either non-targeting control or Pfn-1-specific siRNAs at 50 nM working concentration as previously described [22]. Twenty-four hours after transfection, cells were treated with either a LOPAC compound or DMSO (control) for 48 hours before being analyzed for single cell motility (see above) or Western blot analyses. Total cell lysate was extracted with a modified RIPA buffer [50 mM Tris-HCl (pH 7.5), 150 mM NaCl, 1% Nonidet P40, 0.5% sodium deoxycholate, 0.3% SDS, 2 mM EDTA plus protease and phosphatase inhibitors]. Proteins were separated by SDS-PAGE and transferred to nitrocellulose membranes. For immunoblotting, antibodies specific for Pfn-1 (Novus, NBP1-95847) and α-tubulin (Sigma, T5168) were used at 1∶4000 and 1∶2000, respectively. Bands were visualized with horseradish peroxidase-conjugated secondary antibodies (Jackson Immunoresearch) and an enhanced chemiluminescence reagent.

## Supporting Information

Figure S1 **Profilin-1 induction counterscreen.** Microplates from the primary migration screen were immunostained with a Pfn-1 primary/Cy3 secondary antibody combination and three images acquired on an Arrayscan VTI high content reader using a 20× objective. Nuclei were counterstained with Hoechst 33342. An imaging mask was created based on Hoechst staining. Mean Pfn-1 intensity per cell was measured in the Cy3 channel using an area defined by the nuclear mask, enlarged by three pixels (MEAN_CircAvgIntenCh2). **A.** Trellis plot illustrating screening data from a single replicate run. Whereas no agents caused loss of profilin-1, several compounds elevated profilin-1. **B.** Normal quantiles plot of LOPAC-profilin-1 data. Positive hits caused the data to deviate from normal distribution, necessitating the use of a controls-based hit selection criterion. **C.** Selected scan images illustrating visual appearance of wells with elevated Pfn-1. **D.** Relative positioning of the three imaging fields during the screen adjacent to the exclusion zone and away from the well edge. The field in red represents the position of images shown in **C.**
(TIF)

Figure S2 **Cell cycle profiles.** Archived scan images (acquired with a 10X, 0.5NA objective on the ArrayScan II) from concentration-response confirmation plates were analyzed for DNA content. Total Nuclear Hoechst 33342 fluorescence intensity (representing DNA content) was measured in 4,000 individual cells treated with the indicated concentrations of cytochalasin D (CytD), purvalanol (Purv), or tyrphostin A9 (TyrA9). Graphs show DNA content histograms assembled with the FlowJo software package (Tree Star, Inc., Ashley, OR). The image in **B.** is a montage of an entire well at 10× magnification that illustrates the positioning of imaging fields in relation to exclusion zone and well edge. Data are from a single experiment that has been repeated twice.
(TIF)

Figure S3 **Random 3D collagen invasion assay.** The random collagen invasion assay was performed as described [14]. Briefly, MDA-MB-231 cells were treated with vehicle (DMSO) or 10 µM of either purvalanol or tyrphostin A9. 24 h following initial treatment, cells were re-plated for collagen invasion assay. Collagen-I (Type I Rat Tail; BD Biosciences, San Jose, CA), 10× M199 medium and cells were well mixed and poured into duplicate wells of a 24-well plate. Final collagen and cell concentrations were 2.5 mg/ml and 2×10^6^ cells/ml, respectively. The collagen solution was allowed to polymerize for 30 minutes at 37°C and then overlaid with complete growth medium containing 50 ng/ml EGF and 50 ng/ml 12-O-tetradecanoylphorbol-13-acetate (PMA). Real-time imaging of cells was performed at 10 minute intervals for a total duration of 30 hours. The average invasion speed was scored by frame-by-frame analysis of the centroid positions (x, y) of cell nuclei. 20-40 individual cells were scored in each experiment. Both compounds significantly reduced invasion speed compared with vehicle control. Box, 25th and 75th percentiles; whiskers, 10th and 90th percentiles; line, median. Data are the combined values from two independent experiments comprising a total of 37 (DMSO), 38 (purvalanol) and 40 (tyrphostin A9) individual cells (*p<0.001).
(TIF)

Table S1 **Combined hits from multiparametric cell migration/profilin induction screen.**
(TIF)
