# Supplementary figures and images for: A High-Content, Multiplexed Screen in Human Breast Cancer Cells Identifies Profilin-1 Inducers with Anti-Migratory Activities
Source: PLoS One. 2014 Feb 10;9(2):e88350. doi: 10.1371/journal.pone.0088350 (PMC3919756; doi:10.1371/journal.pone.0088350)

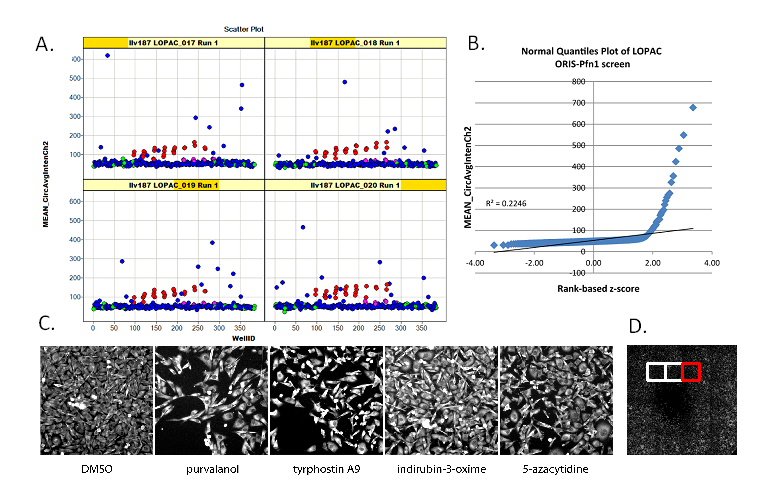

Supplement: Figure S1 — Profilin-1 induction counterscreen. Microplates from the primary migration screen were immunostained with a Pfn-1 primary/Cy3 secondary antibody combination and three images acquired on an Arrayscan VTI high content reader using a 20× objective. Nuclei were counterstained with Hoechst 33342. An imaging mask was created based on Hoechst staining. Mean Pfn-1 intensity per cell was measured in the Cy3 channel using an area defined by the nuclear mask, enlarged by three pixels (MEAN_CircAvgIntenCh2). A. Trellis plot illustrating screening data from a single replicate run. Whereas no agents caused loss of profilin-1, several compounds elevated profilin-1. B. Normal quantiles plot of LOPAC-profilin-1 data. Positive hits caused the data to deviate from normal distribution, necessitating the use of a controls-based hit selection criterion. C. Selected scan images illustrating visual appearance of wells with elevated Pfn-1. D. Relative positioning of the three imaging fields during the screen adjacent to the exclusion zone and away from the well edge. The field in red represents the position of images shown in C. (TIF) [file pone.0088350.s001.tif]

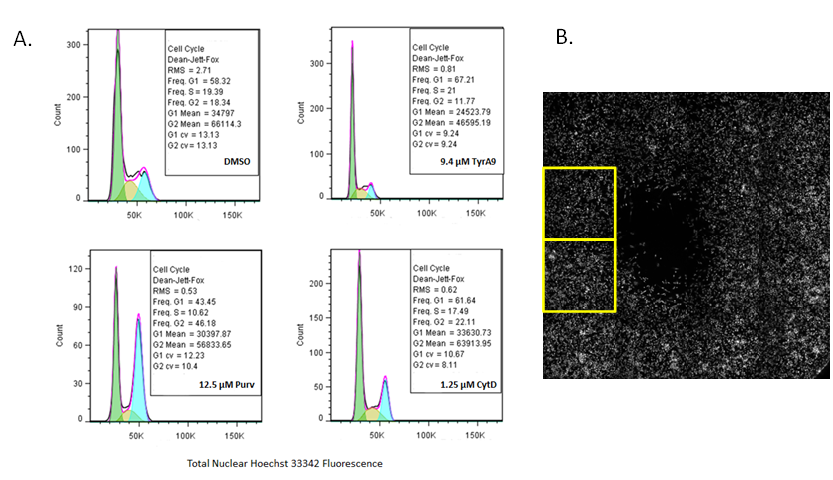

Supplement: Figure S2 — Cell cycle profiles. Archived scan images (acquired with a 10X, 0.5NA objective on the ArrayScan II) from concentration-response confirmation plates were analyzed for DNA content. Total Nuclear Hoechst 33342 fluorescence intensity (representing DNA content) was measured in 4,000 individual cells treated with the indicated concentrations of cytochalasin D (CytD), purvalanol (Purv), or tyrphostin A9 (TyrA9). Graphs show DNA content histograms assembled with the FlowJo software package (Tree Star, Inc., Ashley, OR). The image in B. is a montage of an entire well at 10× magnification that illustrates the positioning of imaging fields in relation to exclusion zone and well edge. Data are from a single experiment that has been repeated twice. (TIF) [file pone.0088350.s002.tif]

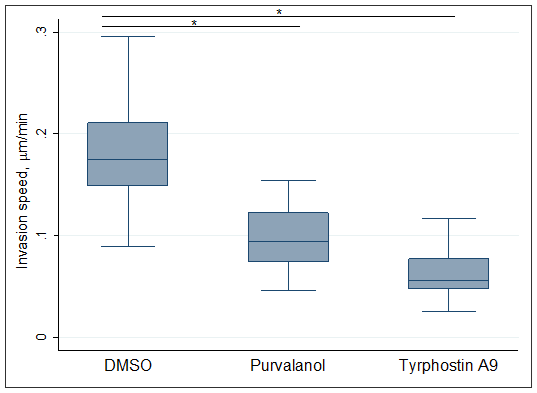

Supplement: Figure S3 — Random 3D collagen invasion assay. The random collagen invasion assay was performed as described [14]. Briefly, MDA-MB-231 cells were treated with vehicle (DMSO) or 10 µM of either purvalanol or tyrphostin A9. 24 h following initial treatment, cells were re-plated for collagen invasion assay. Collagen-I (Type I Rat Tail; BD Biosciences, San Jose, CA), 10× M199 medium and cells were well mixed and poured into duplicate wells of a 24-well plate. Final collagen and cell concentrations were 2.5 mg/ml and 2×10^6^ cells/ml, respectively. The collagen solution was allowed to polymerize for 30 minutes at 37°C and then overlaid with complete growth medium containing 50 ng/ml EGF and 50 ng/ml 12-O-tetradecanoylphorbol-13-acetate (PMA). Real-time imaging of cells was performed at 10 minute intervals for a total duration of 30 hours. The average invasion speed was scored by frame-by-frame analysis of the centroid positions (x, y) of cell nuclei. 20-40 individual cells were scored in each experiment. Both compounds significantly reduced invasion speed compared with vehicle control. Box, 25th and 75th percentiles; whiskers, 10th and 90th percentiles; line, median. Data are the combined values from two independent experiments comprising a total of 37 (DMSO), 38 (purvalanol) and 40 (tyrphostin A9) individual cells (*p<0.001). (TIF) [file pone.0088350.s003.tif]

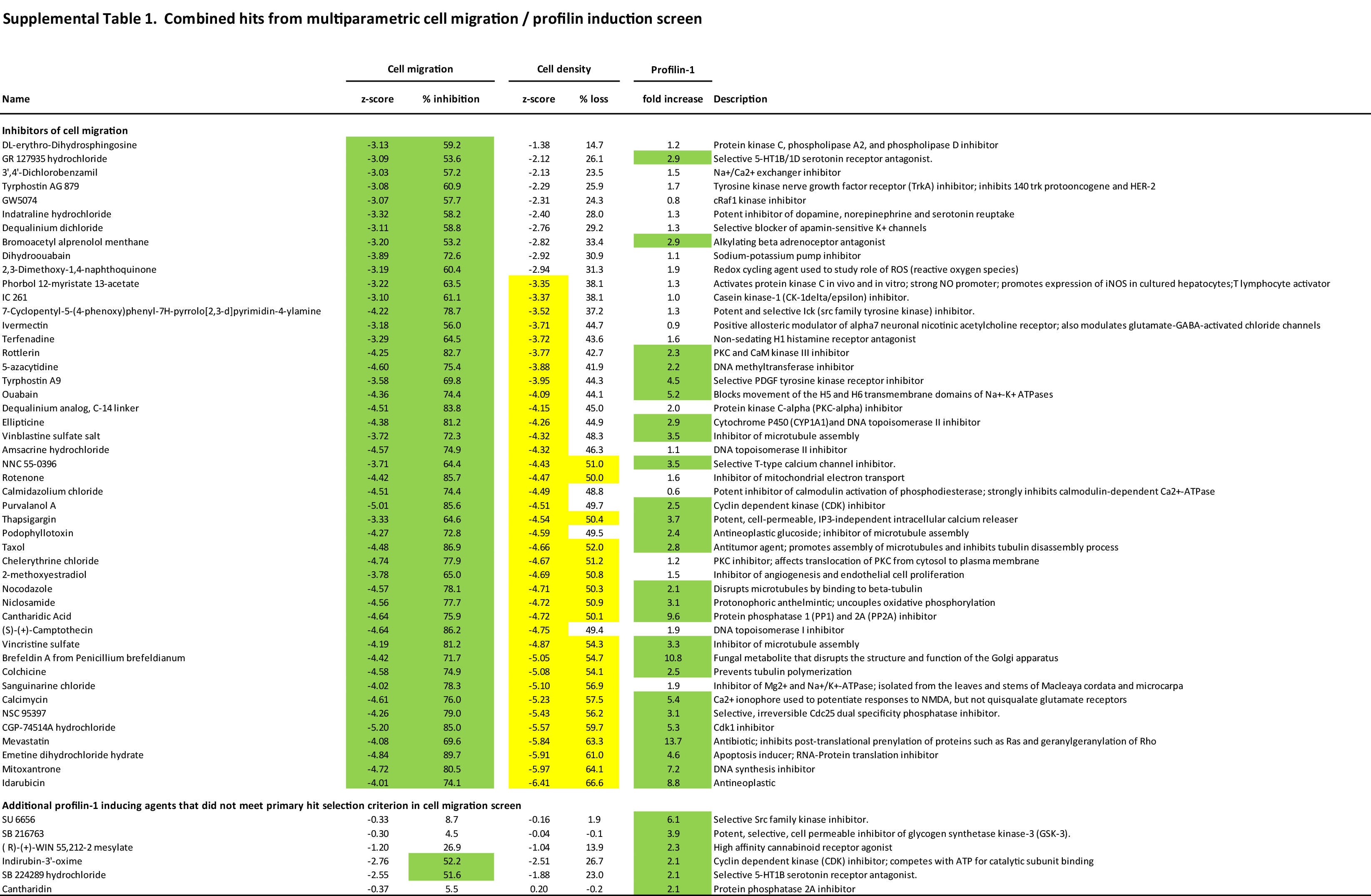

Supplement: Table S1 — Combined hits from multiparametric cell migration/profilin induction screen. (TIF) [file pone.0088350.s004.tif]
